# Comparative Mitochondrial Genome Analysis of *Eligma narcissus* and other Lepidopteran Insects Reveals Conserved Mitochondrial Genome Organization and Phylogenetic Relationships

**DOI:** 10.1038/srep26387

**Published:** 2016-05-25

**Authors:** Li-Shang Dai, Bao-Jian Zhu, Yue Zhao, Cong-Fen Zhang, Chao-Liang Liu

**Affiliations:** 1 College of Life Sciences, Anhui Agricultural University, 130 Changjiang West Road, Hefei 230036, China

## Abstract

In this study, we sequenced the complete mitochondrial genome of *Eligma narcissus* and compared it with 18 other lepidopteran species. The mitochondrial genome (mitogenome) was a circular molecule of 15,376 bp containing 13 protein-coding genes (PCGs), 22 transfer RNA (tRNA) genes, two ribosomal RNA (rRNA) genes and an adenine (A) + thymine (T) − rich region. The positive AT skew (0.007) indicated the occurrence of more As than Ts. The arrangement of 13 PCGs was similar to that of other sequenced lepidopterans. All PCGs were initiated by ATN codons, except for the *cytochrome c oxidase subunit 1* (*cox1*) gene, which was initiated by the CGA sequence, as observed in other lepidopterans. The results of the codon usage analysis indicated that *Asn, Ile, Leu, Tyr* and *Phe* were the five most frequent amino acids. All tRNA genes were shown to be folded into the expected typical cloverleaf structure observed for mitochondrial tRNA genes. Phylogenetic relationships were analyzed based on the nucleotide sequences of 13 PCGs from other insect mitogenomes, which confirmed that *E. narcissus* is a member of the Noctuidae superfamily.

The Ailanthus defoliator *Eligma narcissus* is a member of the family Noctuidae (superfamily Noctuidae), which has spread all over China. This insect pest completes three or four generations every year. It is an important species that is harmful to many plants of economic importance, such as *Ailanthus altissima, Amygdalus persica L., Toona sinensis*, and others^1^. Many studies have been conducted on the biology and chemical pest control of *E. narcissus*^1^,^2^. However, no research has been conducted on its mitochondrial genome.

Insect mitochondrial DNA (mtDNA) has been widely used for species identification and for population genetics and molecular evolution studies due to the low or lack of sequence recombination and its maternal inheritance^3^. In most insects, mtDNA is composed of small, double strands: an L-strand, which is biased in favor of As and Cs, and an H-strand, which has an abundance of Ts and Gs^4^. Moreover, the mtDNA is a relatively conserved circular molecule of 14–19 kb in length^5^ and includes 13 protein-coding genes (PCGs: *atp6, atp8, cox1, cox2, cox3, cob, nad1, nad2, nad3, nad4, nad5, nad6*, and *nad4L*), large and small ribosomal RNA (*rrnL* and *rrnS*) genes, 22 transfer RNA (tRNA) genes and a large non-coding element called the A+T-rich region that contains sequences essential for the initiation of transcription and gene replication^6^.

Lepidoptera (moths and butterflies) is the second largest order in Insecta, accounting for more than 155,000 insect species^7^. Although the superfamily Noctuidae is the largest superfamily in Lepidoptera, only few mitogenomes of Noctuidae are available in GenBank (Table 1). In the present study, we determined the complete mitogenome sequence of *E. narcissus* and compared the nucleotide composition, codon usage, tRNA secondary structure and A+T rich region with those of other lepidopteran species. Furthermore, the concatenated nucleotide sequence of 13 PCGs of *E. narcissus* was used to provide insight into the phylogenetic relationships among lepidopteran superfamilies.

## Results and Discussion

### Genome organization and base composition

**Figure 1:**
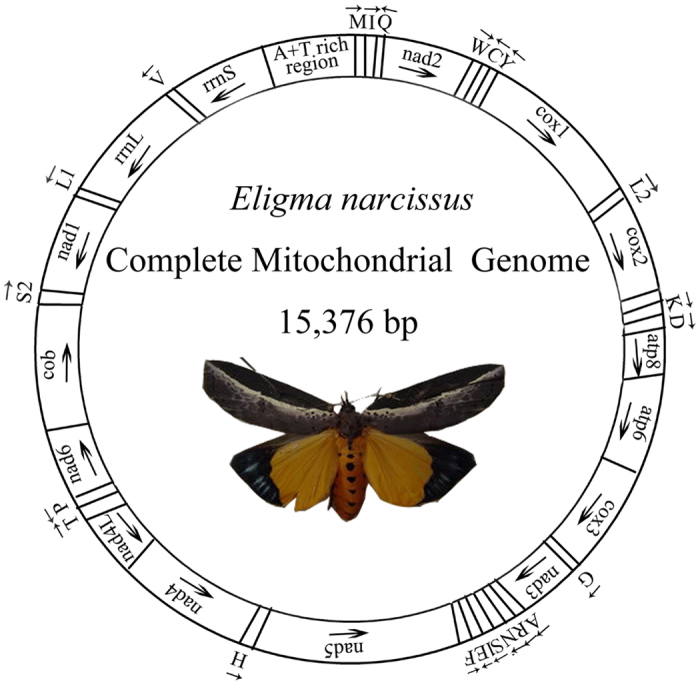
Circular map of the mitogenome of *E. narcissus. cox1, cox2* and *cox3* refer to the *cytochrome c* oxidase subunits. *cob* refers to *cytochrome b. nad1-nad6* refer to *NADH* dehydrogenase components. *rrnL* and *rrnS* refer to ribosomal RNAs. tRNAs are denoted as a one-letter symbol according to the IUPAC-IUB single-letter amino acid codes. Gene names with lines indicate that these genes are located on L strand, whereas the others are located on H strand.

The complete mitogenome of *E. narcissus* is a circular molecule of 15,376 bp in size (Fig. 1), which is bigger than the genomes of *A. selene* (15,236 bp) and *H. armigera* (15,347 bp) but smaller than the genomes of *P. flavescens* (15,659 bp) and *M. sexta* (15,516 bp). The mitogenome of *E. narcissus* contains a remarkably conserved set of 37 genes, including 13 protein-coding genes (PCGs: *atp6, atp8, cox1, cox2, cox3, cob, nad1, nad2, nad3, nad4, nad5, nad6*, and *nad4L*), large and small ribosomal RNA (*rrnL* and *rrnS*) genes, 22 transfer RNA (tRNA) genes and a large non-coding element called the A+T-rich region. The gene order is the same as in other sequenced Noctuidae mitogenomes^8^,^9^,^10^, with a *trnM-trnI-trnQ* order, which is different from the ancestral *trnI-trnQ-trnM* order^11^. The nucleotide composition (A: 40.78%, T: 40.21%, G: 7.68%, C: 11.33%) of the *E. narcissus* mitogenome is biased toward A+T nucleotides (80.99%), which is a higher percentage than that of six other lepidopterans but a lower percentage than that of *M. sexta* (81.79%) (Table 2). The positive AT skew (0.007) indicates the occurrence of more As than Ts, similar to some lepidopterans such as *H. armigera* (0.001)^12^, *O. lunifer* (0.030)^13^, and the Chinese *B. mandarina* (0.057)^14^.

In the mitogenome of *E. narcissus*, there are a total of 34 bp that contain overlapping genes. The 34 bp gene overlap in 11 positions of the *E. narcissus* mitogenome ranges in size from 1 to 8 bp, and the longest overlapping region is present between *trnW* and *trnC*. A total of 192 bp of intergenic spacers are dispersed in 16 regions and range in size from 1 to 52 bp, with the longest spacer present between *trnQ* and *nad2*. The length of the intergenic spacers is considerably longer than that of *A. selene* (137 bp over 13 regions) but shorter than that of *O. lunifer* (371 bp over 20 regions)^9^,^13^.

### Protein-coding genes and codon usage

The 13 PCGs of *E. narcissus* are 11,181 bp in length, accounting for 72.72% of the entire mitochondrial genome. Like other lepidopterans, all 13 PCGs in the *E. narcissus* mitogenome have ATN as their start codon, except for the *cox1* gene, which uses CGA instead. The start codon of the *cox1* is rare in insect mtDNA; the canonical codons TTG, ACG, TTA and TTAG are often reported as the *cox1* start codons^15^,^16^,^17^,^18^. Ten of the 13 PCGs harbor the complete stop codon TAA, whereas the other three possess the incomplete termination codon T for *cox1* and *cox2*, and TA for *nad4* (Table 3). Incomplete stop codons are commonly observed in lepidopteran species^9^,^19^.

**Figure 2:**
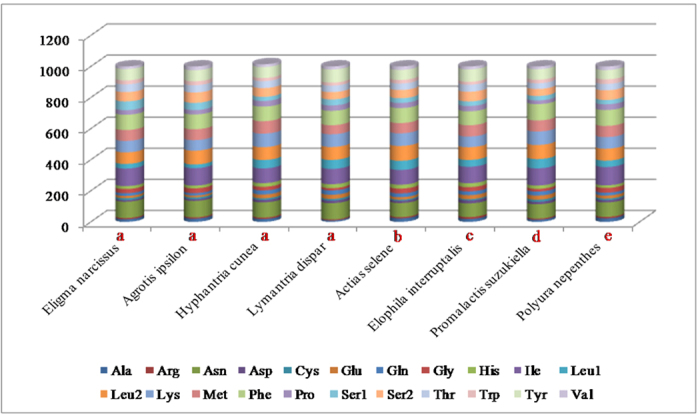
Comparison of codon usage in mitochondrial genomes in Lepidoptera. Lowercase letters (**a–e**) above the species name represent the superfamily that the species belong to ((**a**) Noctuidae, (**b**) Bombycoidea, (**c**) Pyraloidea, (**d**) Gelechioidea, (**e**) Papilionoidea).

**Figure 3:**
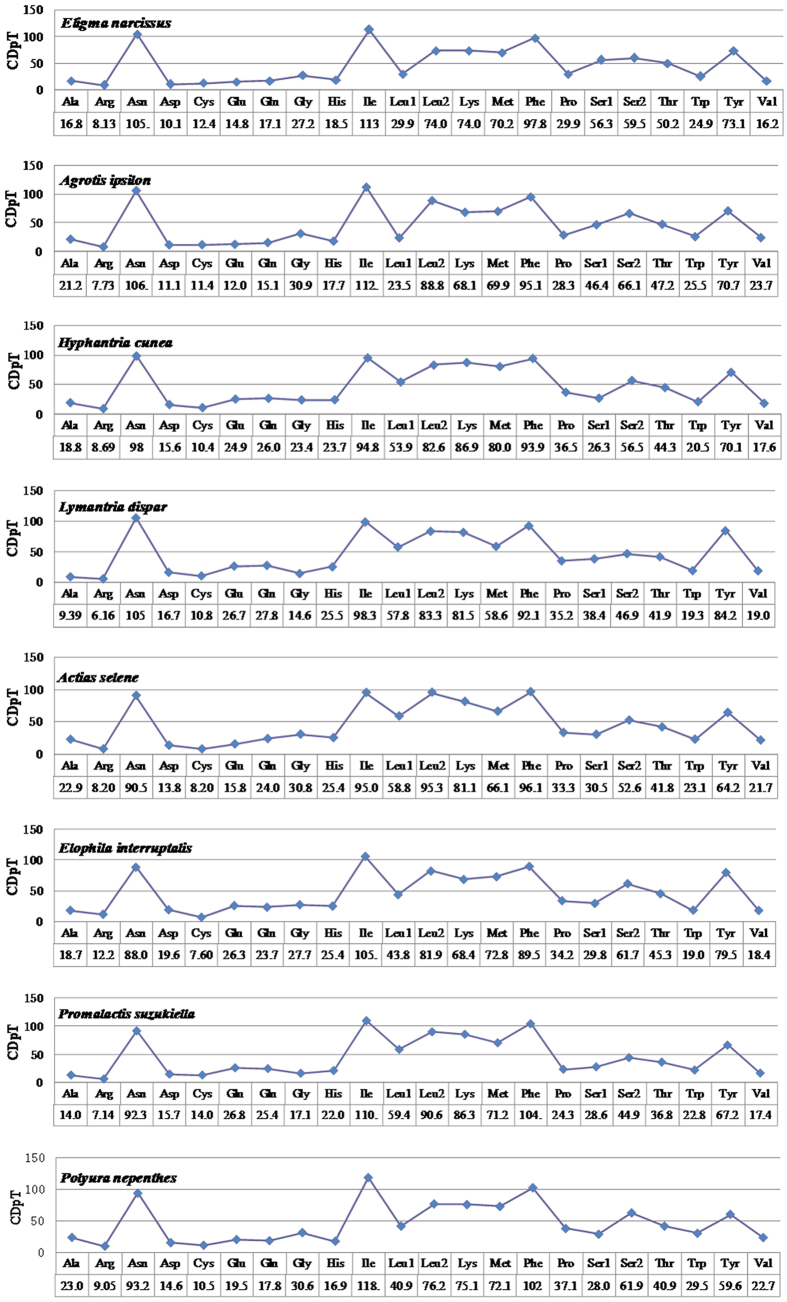
Codon distribution in Lepidoptera. CDspT, codons per thousand codons.

**Figure 4:**
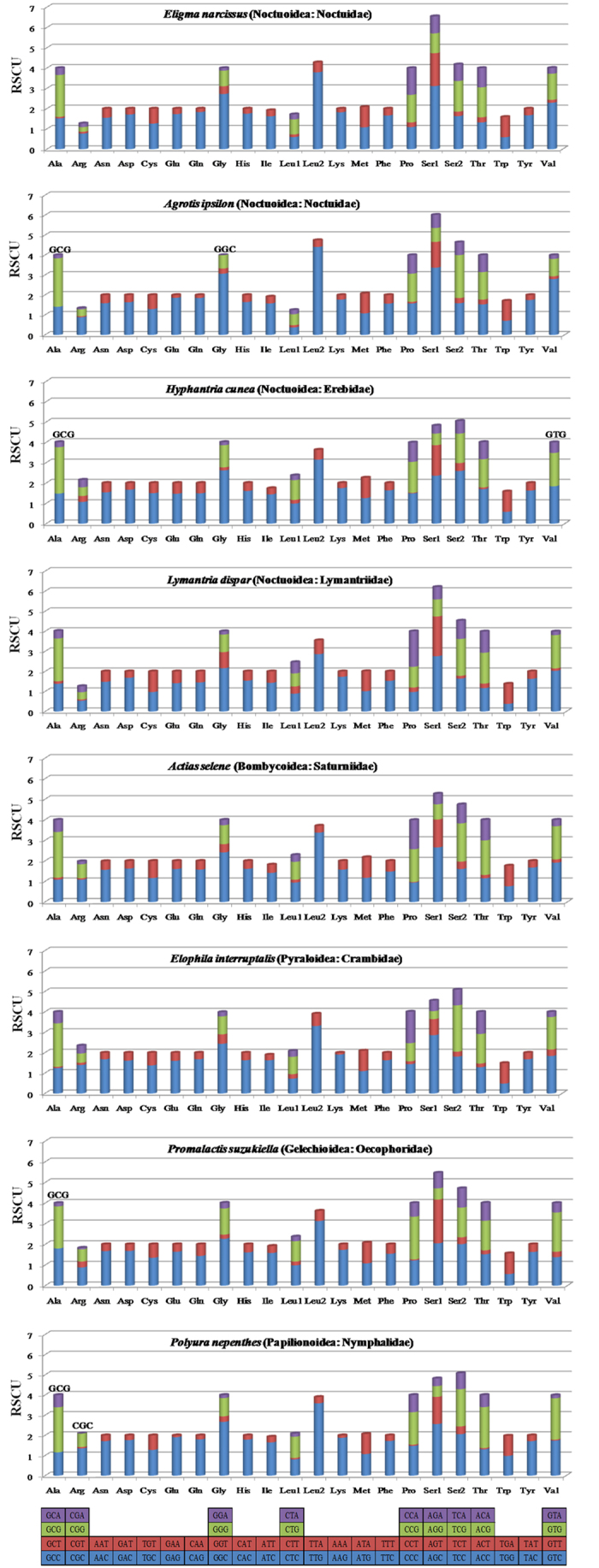
The mitochondrial genome relative synonymous codon usage (RSCU) across five superfamilies in Lepidoptera. Codon families are provided on the X axis. Codons indicated above the bar are not present in the mitogenome.

The 13 PCGs of the *E. narcissus* mitogenome contain 3727 codons in total, which is within the range of 3687 in *H. armigera* and 3742 in *Agrotis ipsilon*. The complete nucleotide sequences of seven lepidopteran insects were downloaded from GenBank to investigate the codon usage among lepidopterans. These mitogenomes are divided into five superfamilies: four species belong to Noctuidae, and the others belong to Bombycoidea, Pyraloidea, Gelechioidea, and Papilionoidea (Fig. 2). We looked at the behavior of codon families in the PCGs and found that *Asn, Ile, Leu, Phe*, and *Tyr* are the most abundant amino acids in the *E. narcissus* mitogenome (Fig. 3). RSCU for Lepidoptera is shown in Fig. 4. All possible codons are present in the PCGs of the *E. narcissus* mitogenome, whereas some codons, such as GCG, GGC, GTG and CGC, are not found in four other species. Previous research indicates that codons with high G and C content are likely not to be favored, a phenomenon that is found in some lepidopteran insects^7^,^20^.

### Ribosomal RNA genes and transfer RNA genes

As with all other insect mitogenome sequences, there were two rRNAs in *E. narcissus* with a total length of 2122 bp. The large ribosomal gene (*rrnL*), located between *trnL1(CUN)* and *trnV,* had a length of 1335 bp, whereas the small one (*rrnS*), located between *trnV* and the A+T- rich region, had a length of 786 bp. The negative AT skew (−0.335) indicated the occurrence of more Ts than As. The A+T content of the two rRNA genes was 84.50%, which was within the range of 82.15% in *O. lunifer* and 85.42% in *M. sexta* (Table 2).

**Figure 5:**
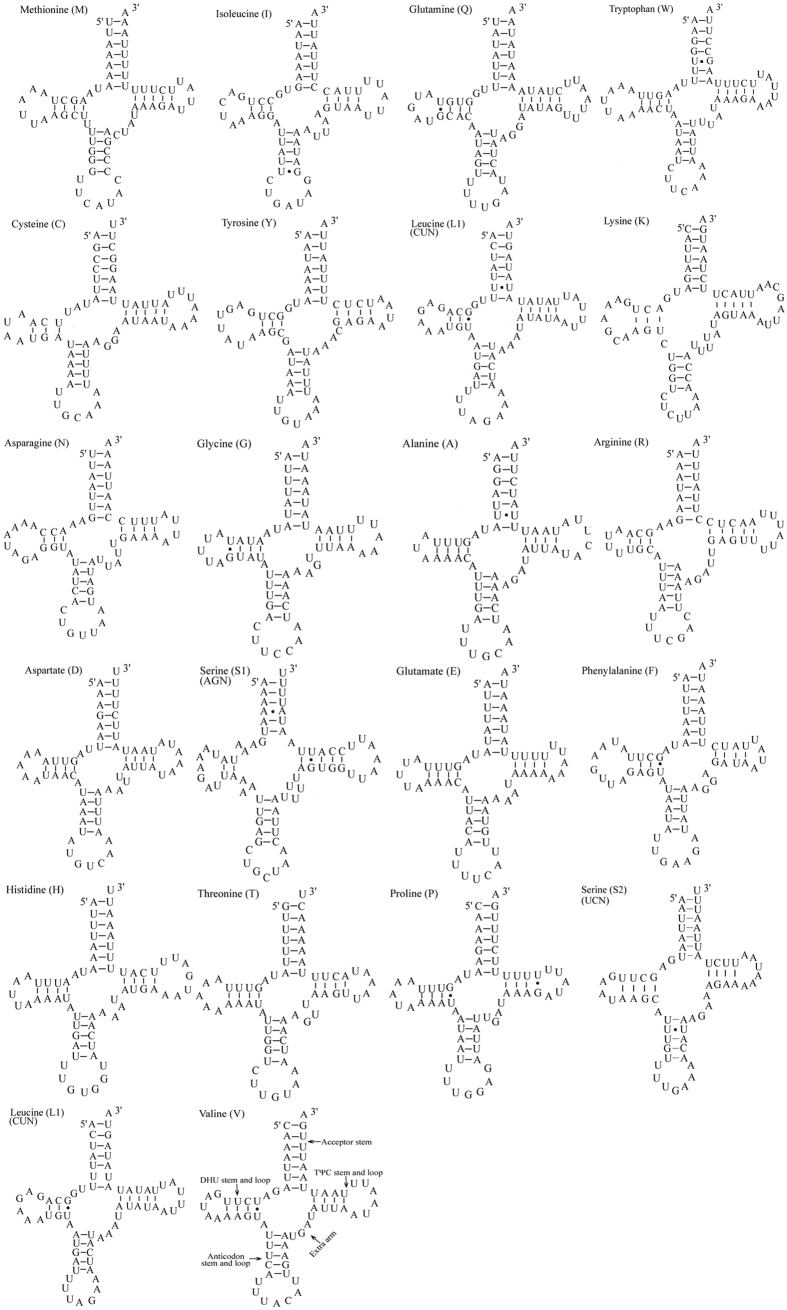
Predicted secondary cloverleaf structures of *E. narcissus* tRNA genes.

The *E. narcissus* mitogenome contained 22 tRNAs interspersed throughout the entire genome and ranging in size from 63 to 71 bp, which comprised 1450 bp of the total mitogenome overall. Of these genes, 14 were encoded by the H-strand, and eight were encoded by the L-strand. The predicted structures of the tRNAs are shown in Fig. 5. The A+T content of the 22 tRNAs was 82.00%, with a positive AT skew (0.013). A total of 11 mismatched base pairs were identified in the tRNAs of *E. narcissus*, three of them being mismatched base pairs (1 A–A and 2 U-U) and eight being G-U wobble pairs. In many insect mitogenomes, the *trnS1* (AGN) gene has an unusual secondary structure lacking a stable stem-loop structure in the DHU arm^9^,^19^; however, we found that all the tRNA genes in *E. narcissus* could be folded into the expected typical cloverleaf secondary structure observed in mitochondrial tRNA genes. All of the secondary structures were drawn using the RNA structure program.

### The A+T-rich region

**Figure 6:**
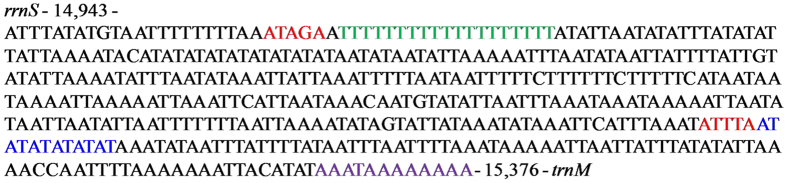
Features present in the A+T-rich region of *E. narcissus*. The motif, poly-T stretch, microsatellite T/A repeat sequences and poly-A stretch are colored in red, green, blue and purple, respectively.

**Figure 7:**
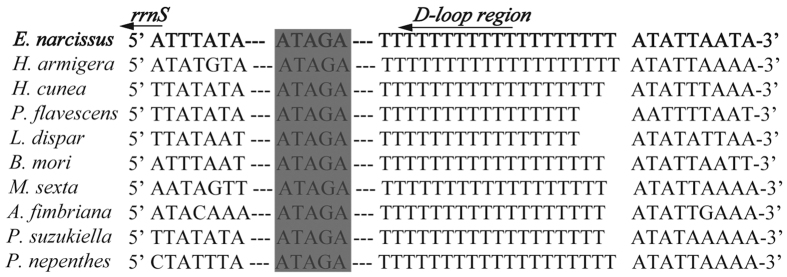
Sequence alignment of the partial *D-loop* region of 10 moth species. The boxed nucleotides indicate the ‘ATAGA’ conserved motif.

The A+T-rich region, known for the initiation of replication in vertebrates and invertebrates, is located between the *rrnS* and *trnM* genes in *E. narcissus*. This region is 434 bp in length, which is longer than the regions in *A. selene* (339 bp) and *M. sexta* (324 bp) but shorter than the regions in Chinese *B. mandarina* (484 bp) and *P. flavescens* (541 bp) (Table 2). The region contains the highest A+T content (96.54%) in the mitogenome. There are some conserved structures observed in the *E. narcissus* A+T-rich region, including the motif ‘ATAGA’ followed by an 19 bp poly-T stretch, the motif ‘ATTTA’ followed by a microsatellite-like (AT) motif^ 6^ and a poly-A element upstream of the *trnM* gene (Fig. 6). The poly-T is located upstream of the *rrnS* 5′-end and is preceded by the ‘ATAGA’ motif, a structural feature that is found in the majority of lepidopteran insects (Fig. 7). Previous research indicates that the poly-T element may be involved in controlling transcription and replication initiation^21^. Two repeat elements are found in the A+T-rich region of the *E. narcissus* mitogenome. Some lepidopteran insects also have repeat elements in the A+T-rich region. The A+T-rich region of *S. longistyla* harbors two repeat elements^22^, whereas the *C. medinalis* and *C. suppressalis* mitogenomes contain a duplicated 25 bp repeat element and a duplicated 36 bp repeat element, respectively^20^.

### Phylogenetic analyses

**Figure 8:**
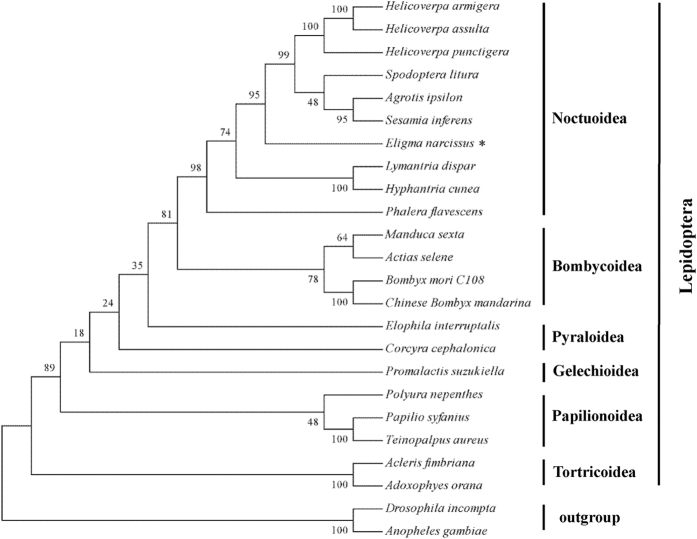
Phylogenetic analysis of lepidopteran insects. The phylogenetic tree was constructed using the maximum likelihood (ML) method, and bootstrap values (1000 repetitions) of the branches were indicated. *D. incompta* (NC_025936) and *A. gambiae* (NC_002084) were used as outgroups.

In this study, the mitogenomes of 18 lepidopteran species representing six lepidopteran superfamilies (Noctuoidea, Bombycoidea, Pyraloidea, Tortricoidea, Geometroidea, Papilionoidea) were downloaded from GenBank. The phylogenetic relationships among the superfamilies of Lepidoptera were reconstructed based on concatenated nucleotide sequences of 13 PCGs by using the maximum likelihood (ML) method (Fig. 8). The phylogenetic analyses show that *E. narcissus* was within Noctuidae. Noctuidae is closely related to Bombycoidea. Although these results do not conflict with other published trees^7^,^23^, more studies of a variety of species are needed to provide further insights into the relationships among Noctuidae species.

## Materials and Methods

### Sample collection and DNA extraction

No specific permits were required for the insect collection necessary for this study. *E. narcissus* larvae were collected in Hefei city, China. Specimens identified as *E. narcissus* were preserved in 100% ethanol and stored at −80 °C. Total genomic DNA was extracted with the Aidlab Genomic DNA Extraction Kit (Aidlab Co., Beijing, China) according to the manufacturer’s instructions. The DNA was examined using 1% agarose gels and was then used for PCR amplification of the complete mitogenome.

### PCR amplification, cloning and sequencing

To amplify the entire mitogenome of *E. narcissus,* nine pairs of primers were designed based on the known mitogenomes of lepidopteran species (Beijing Sunbiotech Co., Ltd., Beijing, China) (Table 4). PCR reactions were carried out in a 50 μl reaction volume, including 5 μl of 10× long Taq buffer (Mg2+ plus), 5 μl of dNTP (20 mM), 1.5 μl of DNA template from a single specimen, 2 μl of each primer (10 μM), 35 μl of sterilized distilled water and 0.5 μl (1 unit) of long Taq (Aidlab Co., Beijing, China). The conditions for PCR amplification were as follows: an initial denaturation for 4 min at 94 °C, followed by 35 cycles of 30 s at 94 °C, 40 s at 46–57 °C (depending on primer combination) and 1–3 min (depending on putative length of the fragments) at 72 °C, as well as a final extension step of 72 °C for 10 min. All PCR reactions were performed in a BIO-RAD thermal cycler.

The above PCR products were resolved by agarose gel electrophoresis (1% w/v) and purified using a DNA gel extraction kit (TaKaRa Co., Dalian, China). The purified PCR fragments were ligated into the T-vector (TaKaRa Co., Dalian, China) and then transformed into competent *Escherichia coli* DH5α. The positive recombinant clone with an insert was sequenced at least three times (Invitrogen Co., Ltd., Shanghai, China).

### Genome assembly and gene annotation

The *E. narcissus* final consensus mtDNA sequence was performed using the Lasergene software package (DNASTAR Inc. Madison, USA). Sequence annotation was performed using the Online Blast Tool of the NCBI web site (http://blast.ncbi.nlm.nih.gov/Blast). The overlapping regions and intergenic spacers between genes were counted manually. The base composition of nucleotide sequences was described by skewness and was measured according to the following formulas: AT skew = [A − T]/[A+T]), GC skew = [G − C]/[G+C]. The A+T content and relative synonymous codon usage (RSCU) values were calculated using MEGA 5.0^24^. Transfer RNA genes were identified using the tRNAscan-SE program software available online at http://lowelab.ucsc.edu/tRNAscan-SE/25. The secondary structures of tRNA genes were analyzed by comparison with the nucleotide sequences of other insect tRNA sequences. The nucleotide sequences of the PCGs were translated on the basis of the invertebrate mtDNA genetic code. Alignments of PCGs from other lepidopteran mitogenome sequences were performed using ClustalX software^26^. The entire A+T-rich region was subjected to a search for tandem repeats using the Tandem Repeats Finder program (http://tandem.bu.edu/trf/trf.html)^27^.

### Phylogenetic analysis

Along with the *E. narcissus* mitochondrial genome, 21 available insect mitogenomes were downloaded from GenBank to illustrate the phylogenetic relationships among lepidopteran insects. The mitogenomes of *Drosophila incompta* (NC_025936)^28^ and *Anopheles gambiae* (NC_002084)^29^ were downloaded and used as outgroups. The nucleotide and putative amino acid regions for each of the 13 PCGs were aligned using ClustalW, as implemented in the program MEGA. To select the conserved regions of the putative amino acids, all alignments were analyzed with the program Gblock 0.91b using default settings^30^. Phylogenetic analysis was conducted using the maximum likelihood (ML) method, as implemented in the MEGA 5.0 program^31^. This method was used to infer phylogenetic trees with 1000 bootstrap replicates. Substitution model selection was also conducted based on the lowest BIC scores (Bayesian Information Criterion) using MEGA 5.0. The mtREV24 + G + F model was the appropriate models for the amino acid sequence dataset.

## Additional Information

**How to cite this article**: Dai, L.-S. *et al*. Comparative Mitochondrial Genome Analysis of *Eligma narcissus* and other Lepidopteran Insects Reveals Conserved Mitochondrial Genome Organization and Phylogenetic Relationships. *Sci. Rep.* **6**, 26387; doi: 10.1038/srep26387 (2016).

## Figures and Tables

**Table 1:** Lepidopteran mitogenomes used in this study.

**Superfamily**	**Family**	**Species**	**Acc. number**	**Refference**
Noctuoidea	Noctuidae	*Eligma narcissus*		This study
		*Agrotis ipsilon*	KF163965	32
		*Helicoverpa armigera*	GU188273	12
		*Helicoverpa assulta*	KR149448	unpublished
		*Sesamia inferens*	NC_015835	unpublished
	Erebidae	*Hyphantria cunea*	GU592049	33
	Notodontidae	*Phalera flavescens*	JF440342	34
	Lymantriidae	*Lymantria dispar*	NC_012893	unpublished
Bombycoidea	Bombycidae	*Bombyx mori*	NC_002355	unpublished
	Saturniidae	*Actias selene*	NC_018133	9
	Sphingidae	*Manduca sexta*	EU286785	35
Pyraloidea	Crambidae	*Elophila interruptalis*	NC_021756	36
	Pyralidae	*Corcyra cephalonica*	NC_016866	unpublished
Tortricoidea	Tortricidae	*Acleris fimbriana*	NC_018754	unpublished
		*Adoxophyes orana*	NC_021396	37
Gelechioidea	Oecophoridae	*Promalactis suzukiella*	KM875542	38
Papilionoidea	Papilionidae	*Papilio syfanius*	NC_023978	39
		*Teinopalpus aureus*	NC_014398	unpublished
	Nymphalidae	*Polyura nepenthes*	NC_026073	unpublished

**Table 2:** Composition and skewness in the lepidopteran mitogenomes.

Species	Size (bp)	A%	G%	T%	C%	A+T %	ATskewness	GCskewness
Whole genome								
* E. narcissus*	15,376	40.78	7.68	40.21	11.33	80.99	0.007	−0.192
* H. armigera*	15,347	40.54	7.69	40.43	11.34	80.97	0.001	−0.192
* O. lunifer*	15,593	40.09	7.56	37.75	14.60	77.84	0.030	−0.318
* P. flavescens*	15,659	40.07	7.87	40.80	11.26	80.87	−0.009	−0.177
* B. mandarina*	15,682	43.11	7.40	38.48	11.01	81.59	0.057	−0.196
* A. selene*	15,236	38.54	8.05	40.37	13.03	78.91	−0.023	−0.236
* M. sexta*	15,516	40.67	7.46	41.11	10.76	81.79	−0.005	−0.181
PCG								
* E. narcissus*	11,190	40.68	8.40	37.72	13.20	78.42	0.038	−0.222
* H. armigera*	11,203	34.25	10.62	45.18	9.95	79.43	−0.138	0.033
* O. lunifer*	11,266	32.47	12.08	43.26	12.19	75.73	−0.142	−0.004
* P. flavescens*	11,206	39.40	8.90	39.56	12.15	78.96	−0.002	−0.154
* B. mandarina*	11,196	42.83	8.26	37.04	11.87	79.87	0.072	−0.179
* A. selene*	11,231	37.93	8.74	39.44	13.89	77.37	−0.020	−0.228
* M. sexta*	11,178	40.40	8.22	39.89	11.49	80.29	0.006	−0.166
tRNA								
* E. narcissus*	1450	41.52	8.00	40.48	10.00	82.00	0.013	−0.111
* H. armigera*	1473	41.41	8.15	40.39	10.05	81.81	0.012	−0.104
* O. lunifer*	1666	41.78	7.32	39.86	11.04	81.63	0.023	−0.202
* P. flavescens*	1474	41.66	7.80	40.64	9.91	82.29	0.012	−0.119
* B. mandarina*	1472	41.78	7.81	39.95	10.46	81.73	0.022	−0.145
* A. selene*	1459	40.37	8.16	40.23	11.24	80.60	0.002	−0.159
* M. sexta*	1470	41.09	8.16	40.68	10.07	81.77	0.005	−0.145
rRNAs								
* E. narcissus*	2122	41.89	5.00	42.60	10.51	84.50	−0.008	−0.355
* H. armigera*	2189	41.75	4.89	43.40	9.96	85.15	−0.019	−0.341
* O. lunifer*	2157	41.96	4.82	40.19	13.03	82.15	0.022	−0.460
* P. flavescens*	2198	41.31	4.73	44.04	9.92	85.35	−0.032	−0.354
* B. mandarina*	2134	43.86	4.78	41.05	10.31	84.91	0.028	−0.366
* A. selene*	2126	39.93	4.99	43.79	11.29	83.73	−0.046	−0.387
* M. sexta*	2168	41.37	4.84	44.05	9.73	85.42	−0.031	−0.335
A+T-rich region								
* E. narcissus*	434	47.47	1.15	49.08	2.30	96.54	−0.017	−0.332
* H. armigera*	328	44.51	1.22	50.61	3.66	95.12	−0.064	−0.500
* O. lunifer*	319	44.5	1.6	48.9	5.0	93.4	−0.047	−0.524
* P. flavescens*	541	42.14	2.22	49.72	5.91	91.87	−0.083	−0.454
* B. mandarina*	484	46.49	2.69	47.93	2.89	94.42	−0.015	−0.036
* A. selene*	339	43.07	5.90	44.84	6.19	87.91	−0.020	−0.024
* M. sexta*	324	45.06	1.54	50.31	3.09	95.37	−0.055	−0.419

**Table 3:** Annotation and gene organization of the *Eligma narcissus* mitogenome.

Gene	Direction	Location	Size	Anticodon	Start codon	Stop codon	Intergenic nucleotides*
*trnM*	F	1–67	67	CAT	–	–	0
*trnI*	F	68–132	65	GAT	–	–	−3
*trnQ*	R	130–198	69	TTG	–	–	52
*nad2*	F	251–1264	1014	–	ATT	TAA	1
*trnW*	F	1266–1334	69	TCA	–	–	−8
*trnC*	R	1327–1394	68	GCA	–	–	2
*trnY*	R	1397–1461	65	GTA	–	–	2
*cox1*	F	1464–2997	1534	–	CGA	T	−1
*trnL2(UUR)*	F	2995–3061	67	TAA	–	–	0
*cox2*	F	3062–3743	682	–	ATG	T	0
*trnK*	F	3744–3814	71	CTT	–	–	7
*trnD*	F	3822–3887	66	GTC	–	–	0
*atp8*	F	3888–4052	165	–	ATC	TAA	−7
*atp6*	F	4046–4723	678	–	ATG	TAA	−1
*cox3*	F	4723–5511	789	–	ATG	TAA	2
*trnG*	F	5514–5578	65	TCC	–	–	0
*nad3*	F	5579–5932	354	–	ATT	TAA	42
*trnA*	F	5975–6039	65	TGC	–	–	2
*trnR*	F	6042–6104	63	TCG	–	–	6
*trnN*	F	6111–6175	65	GTT	–	–	5
*trnS1(AGN)*	F	6181–6251	71	GCT	–	–	−1
*trnE*	F	6251–6315	65	TTC	–	–	4
*trnF*	R	6320–6386	67	GAA	–	–	−2
*nad5*	R	6391–8132	1742	–	ATT	TAA	−2
*trnH*	R	8131–8197	67	GTG	–	–	−2
*nad4*	R	8196–9531	1336	–	ATA	TA	−6
*nad4L*	R	9526–9812	287	–	ATT	TAA	23
*trnT*	F	9836–9899	64	TGT	–	–	0
*trnP*	R	9900–9963	64	TGG	–	–	7
*nad6*	F	9971–10,507	537	–	ATA	TAA	−1
*cob*	F	10,507–11,654	1148	–	ATG	TAA	3
*trnS2(UCN)*	F	11,658–11,723	66	TGA	–	–	30
*nad1*	R	11,754–12,683	930	–	ATT	TAA	4
*trnL1(CUN)*	R	12,688–12,754	67	TAG	–	–	0
*rrnL*	R	12,755–14,089	1335	–	–	–	0
*trnV*	R	14,090–14,155	66	TAC	–	–	0
*rrnS*	R	14,156–14,942	787	–	–	–	0
CR		14,943–15,376	434	–	–	–	–

**Table 4:** List of primers used to amplify the mitogenome of *Eligma narcissus.*

Primer	Sequence (5′ → 3′)
F1	ATAGAATTAAACTATATCCTATA
R1	ATCTAAGAGATGTTCCTACTAT
F2	TACAATTTATCGCTTATAACTCA
R2	TATTAGGAGTTATATATGAGTC
F3	TGAAGTTATGAATATTCAGATT
R3	ATTATTTAGGTGTCGAATTAAA
F4	AGCTGCTAACTTAATTTTTAGT
R4	CTGTTTCAGCTTTAGTTCATTCA
F5	TTACCATTAAAATTATAACATCT
R5	AGATTAATTAATAGATTAATTAG
F6	TCAAATTATTCTAAAATCAATCT
R6	CAAATCAAAGAATAGTTTAATA
F7	CGAAACTAACTCTCTCTCACTC
R7	ATATGTACATATTGCCCGTCGCT
F8	TAGAAACACTTTCCAGTACCTC
R8	ATTTTAAATTATTAGGTGAAATT
F9	ATGAACTAAAATACCGCCAAAT
R9	ATTAATATTGATGAGTTGATTAT
