# Author Correction: Comparative Mitochondrial Genome Analysis of *Eligma narcissus* and other Lepidopteran Insects Reveals Conserved Mitochondrial Genome Organization and Phylogenetic Relationships

**DOI:** 10.1038/s41598-020-63938-0

**Published:** 2020-04-24

**Authors:** Li-Shang Dai, Bao-Jian Zhu, Yue Zhao, Cong-Fen Zhang, Chao-Liang Liu

**Affiliations:** College of Life Sciences, Anhui Agricultural University, 130 Changjiang West Road, Hefei, 230036 China

Correction to: *Scientific Reports* 10.1038/srep26387, published online 25 May 2016

The Data Availability section was omitted from this Article. It should read:

“Genome assembly data for this study are deposited in GenBank under accession code MT232644.”

